# Removal of Arsenate and Chromate by Lanthanum-modified Granular Ceramic Material: The Critical Role of Coating Temperature

**DOI:** 10.1038/s41598-019-44165-8

**Published:** 2019-05-22

**Authors:** Haiyan Yang, Yin Wang, John Bender, Shangping Xu

**Affiliations:** 1 Department of Geosciences, University of Wisconsin—Milwaukee, Milwaukee, WI 53201 USA; 2 Department of Civil and Environmental Engineering, University of Wisconsin—Milwaukee, Milwaukee, WI 53201 USA; 3 Peck School of Arts, University of Wisconsin—Milwaukee, Milwaukee, WI 53201 USA

**Keywords:** Geochemistry, Pollution remediation

## Abstract

The development of economical, low-maintenance, environmentally friendly and effective water filtration techniques can have far-reaching public health, social and economic benefits. In this research, a cost-effective La-modified granular ceramic material made of red art clay and recycled paper fiber was developed for the removal of two major anionic contaminants, As(V) (arsenate) and Cr(VI) (chromate). La modification temperature significantly impacted the resulting composition and properties of the adsorbents, and thus played a crucial role in the adsorbent performance. The La-modified ceramic materials were extensively characterized through scanning electron microscopy (SEM), Brunauer–Emmett–Teller (BET) surface area measurement, thermal gravimetric analysis (TGA), zeta potential measurements, and Fourier-transform infrared spectroscopy (FTIR) analysis. The characterization results suggested that surface coating by LaONO_3_-related compounds was critical for As(V) and Cr(VI) adsorption. At the modification temperature of 385 °C, the adsorption of As(V) and Cr(VI) reached maximum, which were 23 mg/g and 13 mg/g, respectively, under circumneutral conditions that are relevant to various aquatic systems. The adsorption kinetics and isotherm, the influence of pH, ionic strength and coexisting anions on As(V) and Cr(VI) adsorption were investigated to further understand both As(V) and Cr(VI) adsorption behavior. Findings from this research showed that La-modified ceramic material made of recycled paper waste represents a cost-effective adsorbent for anionic contaminant removal under environmentally relevant conditions.

## Introduction

About 1.8 billion people, most of whom live in developing countries, do not have the access to safe drinking water^1^ and the consumption of unsafe drinking water can lead to a wide variety of diseases. For instance, it was estimated that ~50 million people in Asia are exposed to arsenic (As) levels exceeding 50 µg/L and half million out of the 50 million people will die from As related cancers^1^. Additionally, at least four million people are exposed to high concentrations of As in drinking water, primarily rural dwellers consuming water from wells in Latin America^2^. In addition to As, chromium (Cr) is among the most widespread heavy metal pollutants in groundwater, because of the improper disposal of industrial wastes and dissolution of Cr-containing minerals^3^. According to World Health Organization (WHO), improved drinking water supply can reduce the global disease burden by 4%. The development of effective, low-cost, low-maintenance and environmentally friendly water filtration techniques can have far-reaching public health, social and economic benefits.

Adsorption represents a mainline strategy in the removal of chemical and microbial contaminants from drinking water. For most widespread adoption, great efforts have been applied to the development of adsorbents from naturally abundant and/or reusable materials, because of their low cost, simplicity to use, and high efficiency^4–8^. For example, natural minerals such as zeolite usually carry negative surface charges and display high cation exchange capacity, and thus they have been widely used as an inexpensive and yet effective adsorbent for the removal of positively charged contaminants such as heavy metals (e.g., Cd^2+^, Pb^2+^) from water^8,9^. The negative charges of natural minerals (e.g. zeolite, kaolinite, montmorillonite) at environmentally relevant pH conditions (i.e., 5–9)^10,11^, however, make them generally ineffective in the adsorption of anionic contaminants, such as As(V) (arsenate) and Cr(VI) (chromate).

Ceramic materials have gained increasing attention during the past decades for water filtration applications^12,13^. Porous ceramic materials are generally prepared with the use of earth-abundant clay minerals as substrates and organic wastes as pore forming materials (e.g., sawdust, rice husk), and can be fabricated into various shapes (e.g., granule, disk and pot filters). The low-cost and easy-to-use feature makes ceramic-based water filtration a sustainable and affordable treatment technique in developing area^14^. Particularly, recent attempts were made to improve the adsorptive removal of anionic contaminants by amending ceramic materials with metal oxides that provide positive adsorption sites^15,16^. However, the removal efficiency is still unsatisfactory, especially under circumneutral conditions most relevant to water treatment^16^.

In this research, lanthanum (La), a relatively low-cost element (as low as ~$4 per kg of industrial grade) was used as an additive to modify the surface of granular ceramic adsorbents that were made of natural clay and recycled paper fiber. Attempts were then made to quantify the adsorption behavior of the La-modified ceramic material for two major anionic water contaminants, As(V) and Cr(VI). Overall, our results demonstrated that (1) La coating is an effective approach to enhance the adsorption of As(V) and Cr(VI) by ceramic granules, (2) La modification temperature is critical for As(V) and Cr(VI) removal by impacting the composition and surface properties of the adsorbent, and (3) the granular adsorbent with the optimum La coating temperature exhibits efficient removal of As(V) and Cr(VI) under a range of environmental matrices. The material developed in this research can particularly help supply safe drinking water within the developing countries because, in addition to the form of granular adsorbents, the porous ceramic can be fabricated as pot filter, disk and candle shapes, all of which can be used to build low-cost point-of-use (POU) water purification systems^17^.

## Results and Discussion

### As(V) and Cr(VI) adsorption by unmodified and La-modified ceramic granules treated at different temperatures

When La(NO_3_)_3_ was used as precursor, the composition and crystalline structure of La coating could be significantly affected by the coating temperature^18–20^. In this research, ceramic granules were modified by La(NO_3_)_3_, and the mixtures were thermally treated at 300 °C, 385 °C and 800 °C to represent dehydration, formation of LaONO_3_, and formation of La_2_O_3_, respectively. The temperature of 500 °C was also selected to represent the formation of intermediate products during the transformation from LaONO_3_ to La_2_O_3_. Single-point adsorption experiments were then performed to determine the baseline adsorption of As(V) and Cr(VI) by the unmodified ceramic adsorbent, as well as the effects of temperature selected for the thermal treatment step on As(V) and Cr(VI) adsorption by the La-modified ceramic adsorbent.

**Figure 1:**
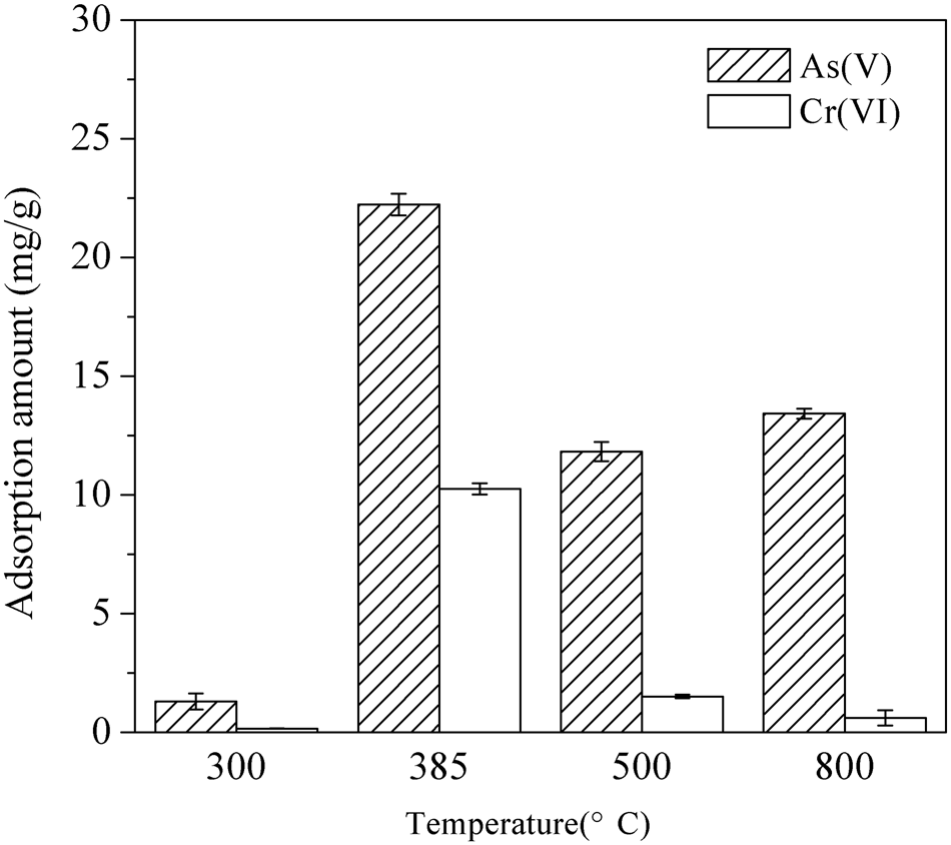
Evaluation of As(V) and Cr(VI) adsorption at room temperature (22 ± 2 °C) on ceramic granules modified with La(NO_3_)_3_ at different temperatures. Solution pH 6.8, As(V) concentration and adsorbent dosage were 30 mg/L and 1.0 g/L, and Cr(VI) concentration and adsorbent dosage were 10 mg/L and 0.5 g/L. The contact time was 24 h. Error bars represent one standard deviation from triplicate experiments.

Our results showed that the unmodified ceramic granules had negligible adsorption for both As(V) and Cr(VI) (data not shown). For the ceramic granules treated at 300 °C, As(V) adsorption was ~1.5 mg/g and the adsorption of Cr(VI) was negligible (Fig. 1). The adsorption of As(V) and Cr(VI) reached maximum (22.2 ± 0.4 mg/g for As(V) and 10.3 ± 0.2 mg/g Cr(VI), respectively) at 385 °C. Further increase in thermal treatment temperature to 500 °C and 800 °C resulted in significantly lower As(V) and Cr(VI) adsorption. Since As(V) and Cr(VI) adsorption by the unmodified ceramic granules was negligible, our results showed that 1) La-modification may be primarily responsible for As(V) and Cr(VI) adsorption; and 2) maximum adsorption amounts were obtained at the thermal treatment temperature of 385 °C for the La modification step.

### Characterization of La-modified ceramic materials

To elucidate the critical role of La coating temperature in the composition and properties of the resulting adsorbents, ceramic granules modified at a series of temperatures (300, 385, 500 and 800 °C) were extensively characterized through scanning electron microscopy (SEM) imaging, Brunauer-Emmett-Teller (BET) surface area measurement, La loading content quantification, Thermogravimetric analysis (TGA), zeta potential measurement and Fourier transform infrared spectroscopy (FTIR) analysis.

**Figure 2:**
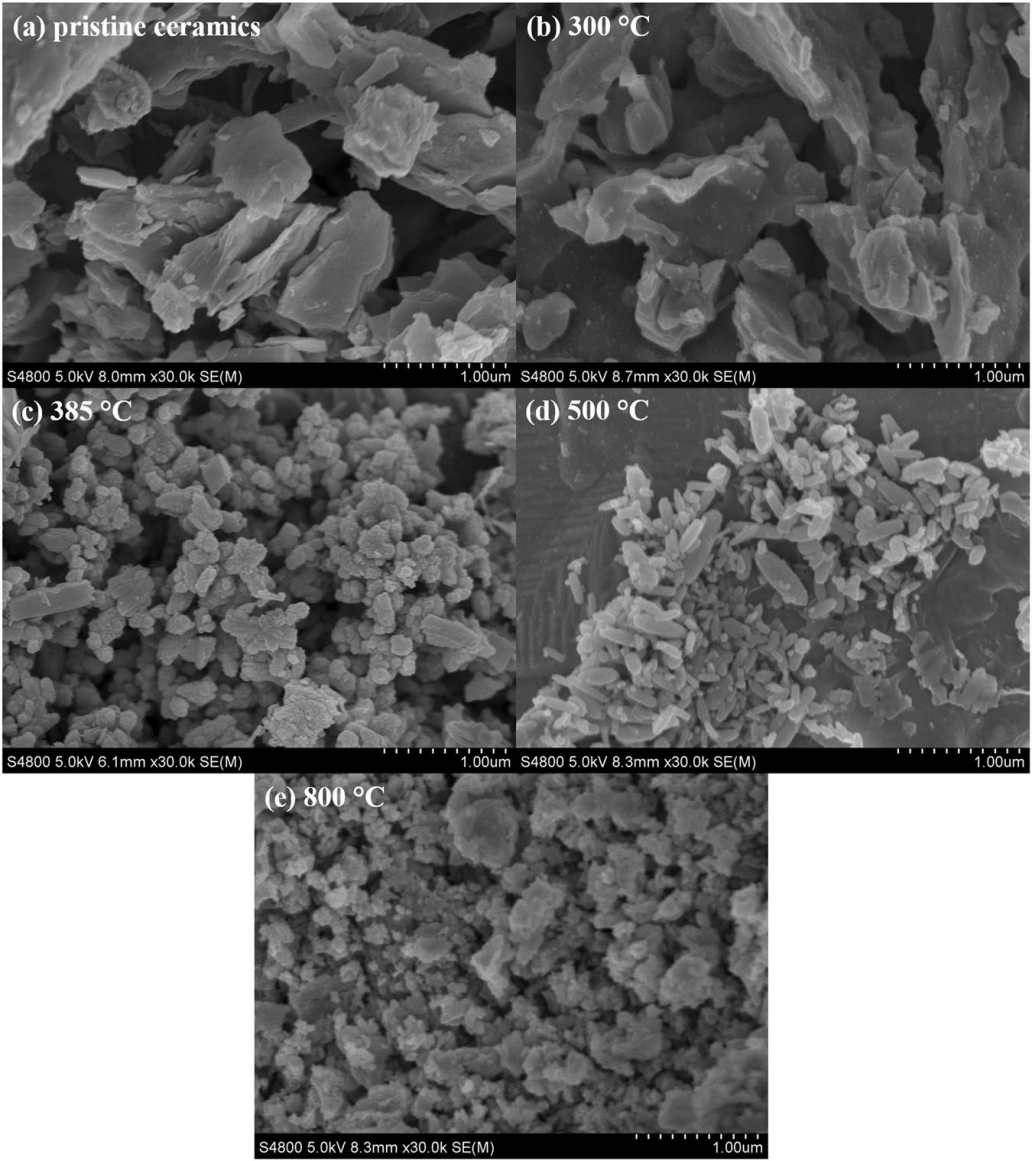
SEM images of granular ceramic materials (**a**) without and with La modification at (**b**) 300, (**c**) 385, (**d**) 500 and (**e**) 800 °C.

SEM images of the unmodified and La-modified ceramic material were obtained to examine their surface morphology (Fig. 2). The surface of the ceramic materials was dominated by micrometer scale plate-shaped structures. The surface of La-modified ceramic materials that were thermally treated at 300 °C appeared similar as the surface of the unmodified ceramic materials. The surface of La-modified ceramic granules that were thermally treated at 385, 500 and 800 °C, however, was covered by high densities of fine particles, indicating the successful coating of La on ceramic surfaces.

The measurement of BET surface area also showed that the unmodified and 300 °C La-modified ceramic materials had similar surface areas (2.79 and 2.65 m^2^/g, respectively), both of which were lower than the surface area of La-modified ceramic materials that were thermally treated at 385 °C and above (SI Table S1). This increase in BET surface area could be attributed to the fine La-containing particles coated on the surface of the ceramic granules. The sorption amounts of As(V) and Cr(VI) were normalized by the surface area of La-coated materials (Fig. S1), and the La-coated material treated at 385 °C showed the highest sorption amount, which was consistent with those normalized by weight (Fig. 1). Results suggested that the enhanced surface area might play a minor role in the enhanced sorption capacity of the La-coated material treated at 385 °C.

Based on acid digestion, the quantity of La extracted from unmodified ceramic materials was below detection limit. SI Table S1 showed that for the La-modified ceramic materials that were thermally treated at 300 °C, the fraction of La was less than 2% (by weight) whereas amount of La increased sharply to 20.4% at 385 °C and remained constant at higher temperatures of 500 and 800 °C. Results were consistent with the morphology and surface area measurements, showing that La modification was successful at temperature ≥385 °C.

**Figure 3:**
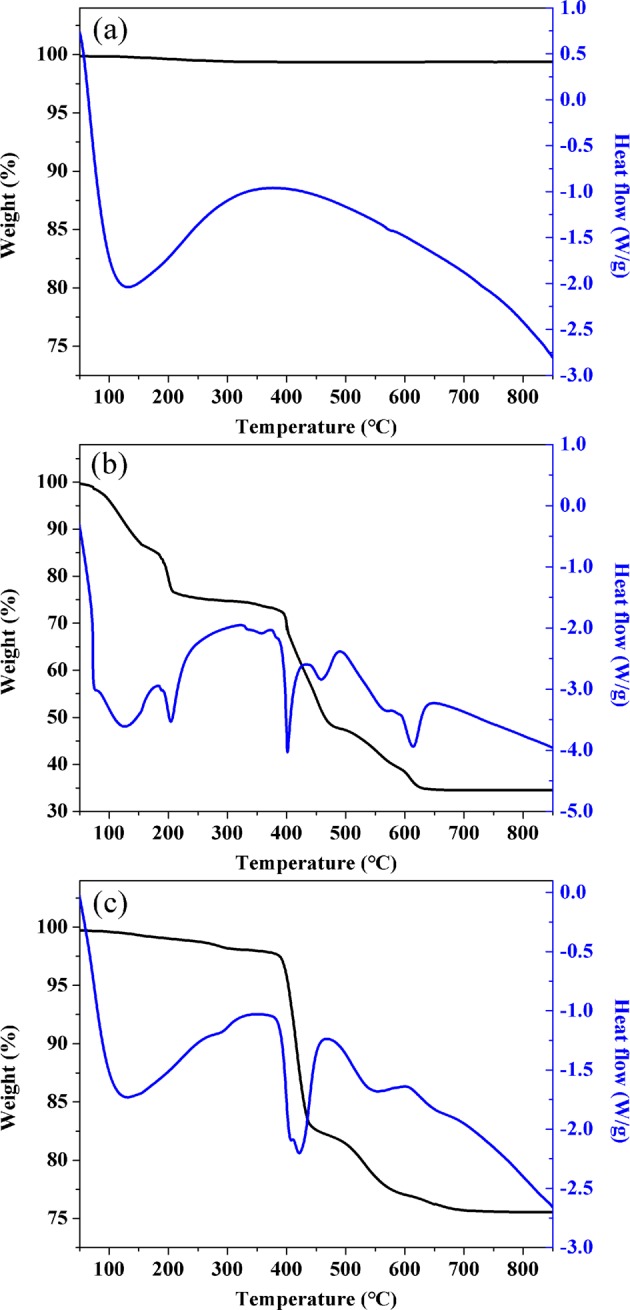
TGA of (**a**) ceramic granules alone, (**b**) La(NO_3_)_3_ and (**c**) ceramic granules treated with La(NO_3_)_3_.

To further determine the composition and structure of La surface coating, TGA was performed for La(NO_3_)_3_·6H_2_O, unmodified ceramic materials and La-modified ceramic materials. For the unmodified ceramic materials, negligible weight loss was observed during thermal heating process (Fig. 3a), indicating that ceramic material was stable during thermal treatment as high as 800 °C. As shown in Fig. 3b, there were four weight loss steps in the TGA curve of La(NO_3_)_3_·6H_2_O amounting from 100% to 74.7%, 74.7% to 50.4%, 50.4% to 34.5% and stable of 34.5%. They corresponded to dehydration, formation of LaONO_3_, decomposition to intermediate components (e.g. La_3_O_4_NO_3_, LaO_2_CO_3_), as well as the conversion from intermediate components to La_2_O_3_, respectively. These weight loss steps and the corresponding temperatures were in excellent agreement with the dehydration and chemical transformation steps observed in previous studies^19,20^_._ The TGA weight loss curve for the La-modified ceramic materials (Fig. 3c) was consistent to that of La(NO_3_)_3_·6H_2_O alone (Fig. 3b). The TGA results indicated that the La compounds coated on the surface of the ceramic materials underwent similar thermal transformation as La(NO_3_)_3_·6H_2_O.

For the La-modified ceramic materials that were treated at 300 °C, the resulting La coating on the surface was likely La(NO_3_)_3,_ which could be easily removed through water rinsing due to its high solubility. This could explain the minimal change of surface morphology, the low La content extracted using diluted HNO_3_, and the lack of adsorption capacity for As(V) and Cr(VI) by the La-modified materials treated at 300 °C.

When the La-modified ceramic materials were treated at 385 °C, the resulting surface coating was predominantly LaONO_3_ and related ligand exchange products in water (e.g., LaOOH). This form of coating was stable and exhibited high affinity for the adsorptive removal of As(V) and Cr(VI). Thermal treatment at higher temperatures transformed LaONO_3_ into intermediate La compounds such as La_2_O_2_CO_3_ and finally to La_2_O_3_ (800 °C). These transformations did not lead to any measurable loss of La content, but significantly lowered the adsorption capacity for As(V) and Cr(VI) (Table S1 and Fig. 1).

**Figure 4:**
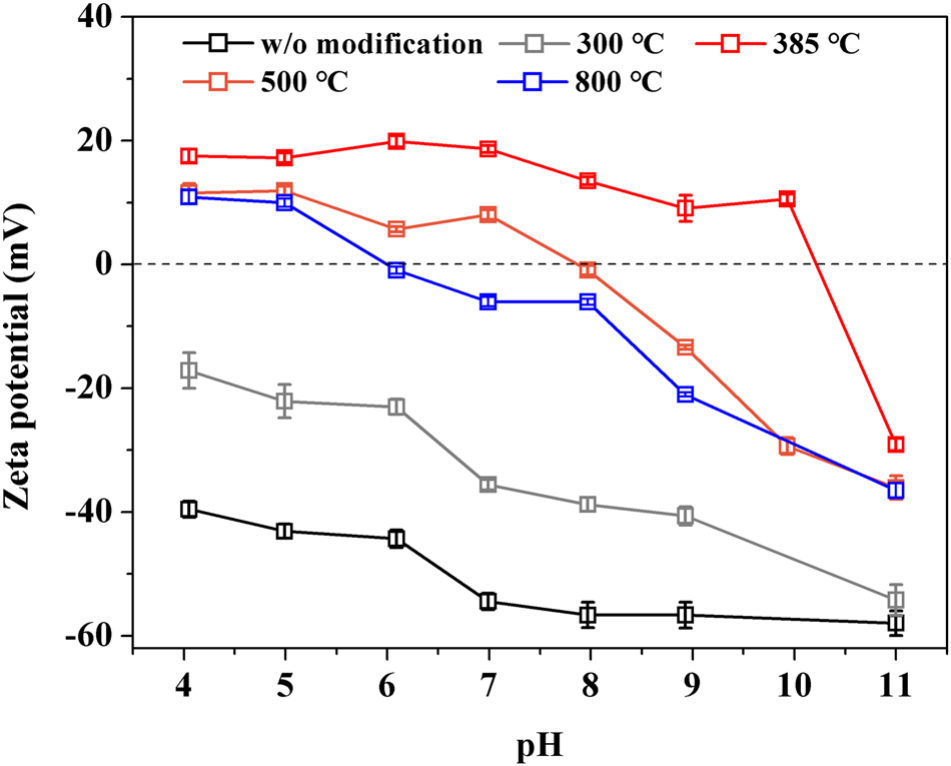
Zeta potential as a function of pH for La-modified ceramic materials treated at different firing temperatures.

Surface charge of unmodified and La-modified ceramic adsorbents could also be closely related to their adsorption behavior for As(V) and Cr(VI), the speciation of which changes dramatically with pH^21,22^. Zeta potential of unmodified and La-modified ceramic particles prepared at different thermal treatment temperatures were measured as a function of pH and present in Fig. 4. For the unmodified ceramic adsorbent, the zeta potential was very negative (<−40 mV) under relatively acidic conditions. The zeta potential dropped by ~15 mV as pH increased from 4 to 11. The highly negative charges on the surface of the unmodified ceramic materials would lead to a repulsive interaction between the negatively charged As(V) (e.g., H_2_AsO_4_^−^, HAsO_4_^2−^) and Cr(VI) (e.g., HCrO_4_^−^, CrO_4_^2−^) ions and prevent their adsorption. This is consistent with the negligible adsorption of As(V) and Cr(VI) by the unmodified ceramic granules.

Compared to the unmodified ceramic material, surface coating by La compounds led to less negative surface charge at all pH conditions as well as a higher point of zero charge (pH_PZC_) (Fig. 4). It is well known that La compounds tend to be positively charged even under basic conditions^23^. Particularly, the surface of La-modified ceramic materials treated at 385 °C was positively charged within the pH range of 4–10. The positive charge was probably caused by the dissociation of NO_3_^−^ from LaONO_3_ and/or the protonation of the associated ligand exchange products (e.g., LaOOH). As the main species of As(V) and Cr(VI) ions under environmentally relevant pH conditions (5.5–8.5) were all negatively charged, the positively charged sites created by La surface coating could facilitate the adsorption of both Cr(VI) and As(V) anions through electrostatic attraction^24,25^. It is interesting to note that the La-modified ceramic materials treated at temperatures higher than 385 °C displayed lower zeta potential values and this observation is consistent with their lower adsorption capacity for both As(V) and Cr(VI).

**Figure 5:**
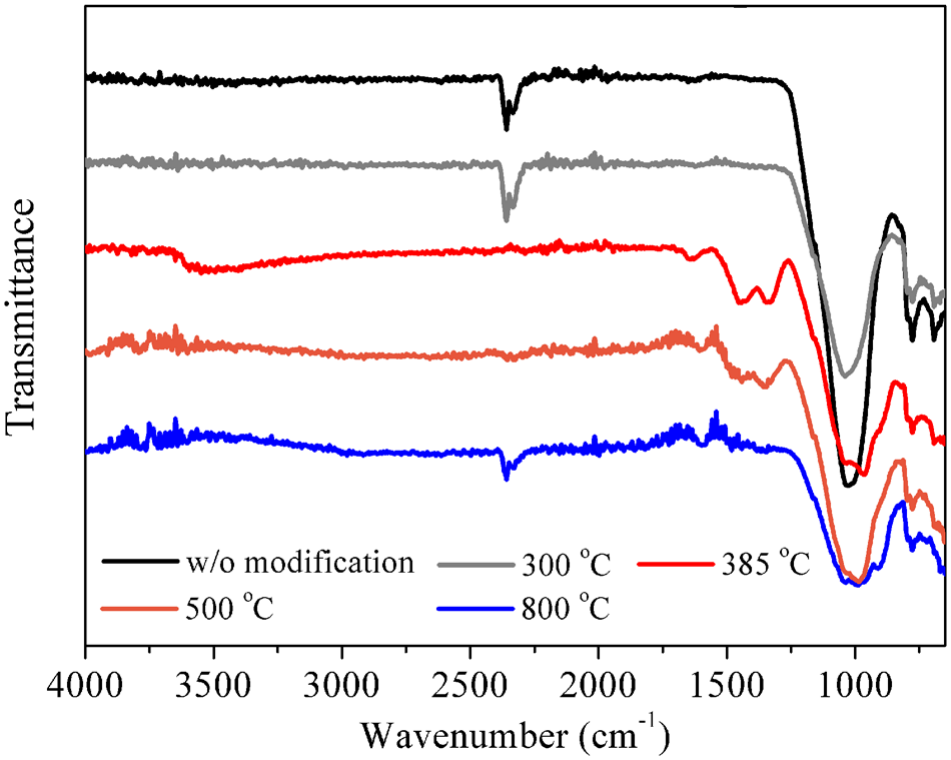
FTIR spectra of La-modified ceramic materials treated at different firing temperatures.

FTIR spectra for unmodified and La-modified ceramic granules at different firing temperatures were measured to gain insights into the surface functional groups on the La-coated ceramic materials and their potential relationship with As(V) and Cr(VI) adsorption. As shown in Fig. 5, the band at 1030 cm^−1^, ascribed to concerted (Si-O-Si) stretches^26^, was observed for ceramic granules both before and after La modification. Unlike the narrow and sharp peak for Si-O-Si stretch on the pristine ceramic surface, there existed a broader and less intense peak around 1000 cm^−1^ for all examined La-modified ceramic materials, which was assigned to the combination bands of La-O fundamental vibrational modes^27^ and Si-O-Si stretch. By comparing FTIR spectra for La-modified ceramic materials among different firing temperatures, significant new peaks at 3554, 1450 and 1300 cm^−1^ were observed for La-modified adsorbent at 385 °C, which corresponded to O-H stretching group of La (hydr)oxide^28^, stretching vibration of H-O-H and vibration mode of NO_3_^−^, respectively. It is consistent with the observation of the main La compound (LaOOH/LaONO_3_) at 385 °C from TGA profile. It is worth noting that these peaks were reduced in the sample modified at 500 °C and they disappeared in the sample modified at 800 °C, indicating significant alteration of surface functional groups during the high temperature treatment steps. The maximum As(V) and Cr(VI) adsorption by the La-modified ceramic adsorbents treated at 385 °C could thus be related to the functional groups of LaOOH/LaONO_3_ that were involved in the sorption process. Previous studies also reported that hydroxyl group induced by La modification played a dominant role in anions adsorption by La-modified red mud and alumina^29,30^.

Overall, the characterization results suggested distinct composition and properties of La-modified ceramic granules treated at different temperatures. Since the adsorbent treated at 385 °C exhibited most favorable features for As(V) and Cr(VI) adsorption, detailed batch experiments were performed to further investigate the adsorption behavior of As(V) and Cr(VI) by the La-modified ceramic granules treated at 385 °C in the following section.

### As(V) and Cr(VI) adsorption on La-modified ceramic granules at 385 °C

#### Adsorption kinetics and isotherms of As(V) and Cr(VI)

**Figure 6:**
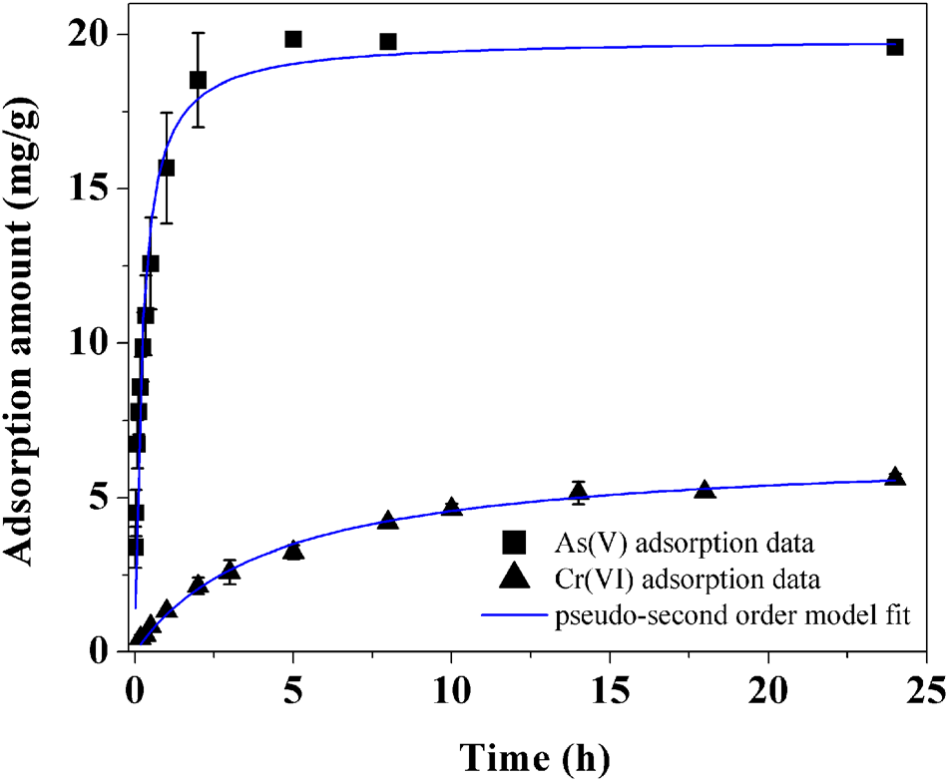
Adsorption kinetics for As(V) and Cr(VI) on La-modified granular ceramic materials treated at 385 °C. Solution pH 6.8, As(V) concentration and adsorbent dosage were 20 mg/L and 1.0 g/L, and Cr(VI) concentration and adsorbent dosage were 3 mg/L and 0.5 g/L, respectively. Error bars represent one standard deviation from triplicate experiments.

Figure 6 shows the adsorption kinetics of As(V) and Cr(VI) by the La-modified ceramic granules treated at 385 °C. Both kinetics curves exhibited a rapid initial uptake and the adsorption plateaued within ~24 h. The adsorption kinetics of As(V) observed in this research was faster than previously reported results while the adsorption kinetics of Cr(VI) was comparable to Cr(VI) adsorption by other reported La-amended adsorbents^29,31^.

Both pseudo-first order and pseudo-second order kinetic models were used to fit the As(V) and Cr(VI) adsorption kinetics data. The two models are expressed in Eqs (1) and (2), respectively^32^.

1 $$q={q}_{e}(1-{e}^{-{k}_{1}t})$$

2 $$q={(\frac{1}{{q}_{e}}+\frac{1}{{k}_{2}{{q}_{e}}^{2}}{t}^{-1})}^{-1}$$

where *q*_*e*_ and *q* stand for the quantities of adsorbed contaminant (mg/g) at equilibrium and at time *t* (h), respectively, and *k*_1_ (h^−1^) and *k*_2_ (g·mg^−1^·h^−1^) represent the rate constants for pseudo-first order and pseudo-second order kinetic models, respectively. Comparison of correlation coefficients (*r*^2^) (Table S2) showed that both As(V) and Cr(VI) adsorption kinetics could be better described with the pseudo-second order kinetic model, which corresponded to a chemisorption process^32^.

**Figure 7:**
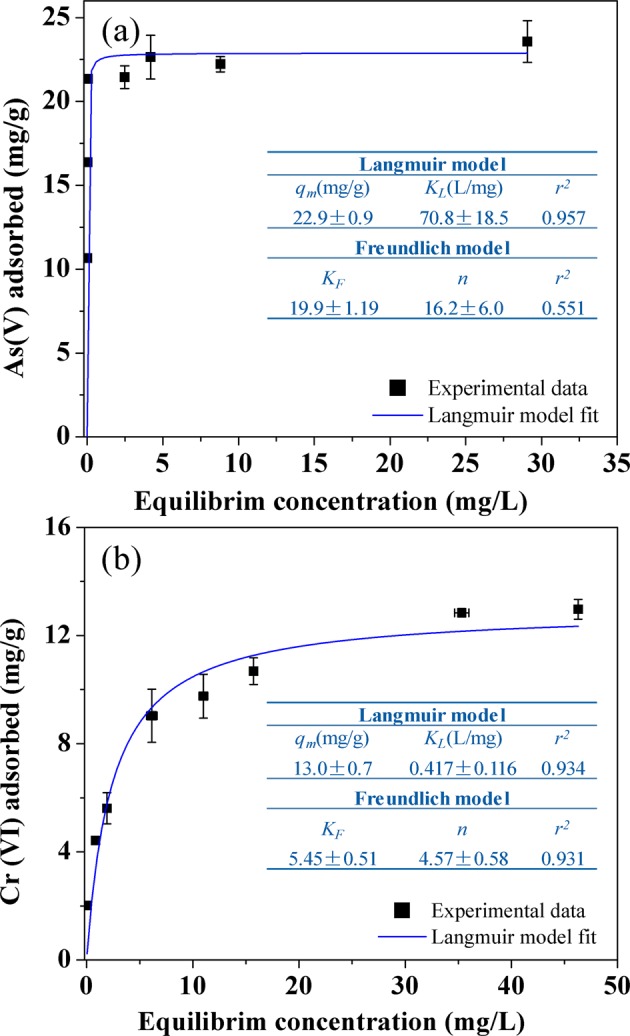
Adsorption isotherms for (**a**) As(V) and (**b**) Cr(VI) on La-modified ceramic granules treated at 385 °C and their Langmuir model isotherm fitting parameters. Solution pH 6.8, the contact time was 24 h, and adsorbent dosages were 1.0 g/L for As(V) and 0.5 g/L for Cr(VI). Error bars represent one standard deviation from triplicate experiments.

As(V) and Cr(VI) adsorption isotherms are presented in Fig. 7. Compared to Cr(VI), the adsorption of As(V) by the La-modified ceramic materials increased more sharply at low aqueous concentrations, indicating a higher affinity of the adsorbents with As(V) than that with Cr(VI). The Langmuir and Freundlich equations were employed to describe the adsorption isotherms in the figure, according to Eqs (3) and (4), respectively^33,34^.

3 $${q}_{e}=\frac{{q}_{m}b{C}_{e}}{1+b{C}_{e}}$$

4 $${q}_{e}=k{C}_{e}^{1/n}$$

where *C*_*e*_ (mg/L) is equilibrium concentration of contaminants in solution, and *q*_*e*_ (mg/g) represents the amount of adsorbed contaminants on unit mass of adsorbent. In Eq. (3), *b* is the Langmuir affinity constant related to energy of adsorption^35^. For the Freundlich model (Eq. (4), *k* signifies the adsorption affinity, while *n* is an indicator related to the heterogeneity of the adsorbent surface.

For both As(V) and Cr(VI) adsorption, Langmuir was more suitable to describe adsorption isotherm (Fig. 7) and the estimated adsorption capacities for As(V) and Cr(VI) were 22.9 ± 0.9 mg/g and 13.0 ± 0.7 mg/g, respectively, calculated from the Langmuir equation. Compared to some reported low-cost or clay/ceramic-based adsorbents, the As(V) adsorption capacity of La-modified ceramic material in this study was 511% and 148% higher than La-impregnated silica gels and Fe-modified ceramic materials, respectively (Table S3)^16,36^. The Cr(VI) adsorption capacity was at least 56% higher than those of bituminous coal and modified clay adsorbents reported previously^37–39^. The La-modified ceramic materials developed in this research can thus serve as a sustainable, low-cost and effective adsorbent for the removal of As(V) and Cr(VI) from drinking water sources.

#### Effects of initial solution pH

**Figure 8:**
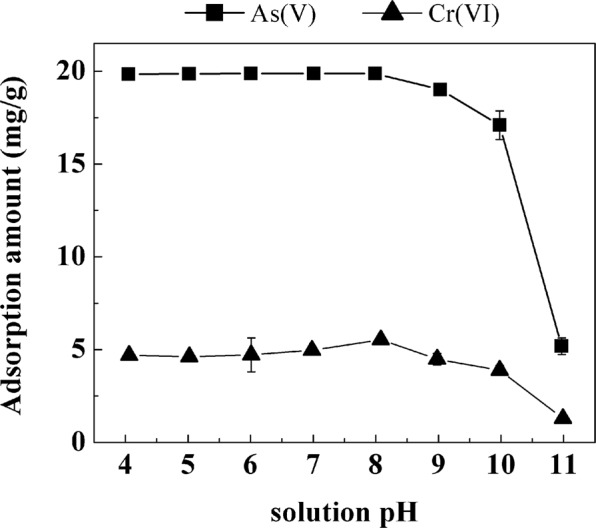
Effect of initial solution pH on As(V) and Cr(VI) adsorption on La-modified ceramic granules. As(V) concentration and adsorbent dosage were 20 mg/L and 1.0 g/L, and Cr(VI) concentration and adsorbent dosage were 3 mg/L and 0.5 g/L. The contact time was 24 h. Error bars represent one standard deviation from triplicate experiments.

To further understand anion adsorption on La-modified ceramic material, the variation of As(V) and Cr(VI) adsorption was examined under a range of initial pH conditions (4–11). As shown in Fig. 8, nearly complete removal of As(V) (>98%) was achieved by La-modified ceramic granules in the pH range from 4–8; further increasing the pH gradually decreased the adsorbent performance and the removal of As(V) declined to ~27% at pH 11. Similar observation was also found for Cr(VI) adsorption that removal of Cr(VI) decreased significantly with pH increasing from 9 to 11. Trends of both As(V) and Cr(VI) adsorption on La-modified ceramic granules as a function of pH were consistent with anionic contaminant adsorption on other La-based adsorbents^31,40–42^. The dominant As(V) and Cr(VI) species in the solution were all anions under the tested pH range (4–11)^21,22^. The lower adsorption of As(V) and Cr(VI) at high pH may be attributed to an increased repulsion between the negatively charged anionic pollutants and more negatively charged surface sites and/or the change of surface sites at high pH conditions that were less favorable to form surface complexes with the anionic pollutants^31,43,44^. The measurement of pH_PZC_ of La-modified ceramic adsorbent at 385 °C (Fig. 4) was also in good agreement with the sharp decrease of As(V) and Cr(VI) adsorption at pH > pH_PZC_ (pH 11) where La-modified ceramic adsorbent was negatively charged.

#### Effects of ionic strength and coexisting anions

**Figure 9:**
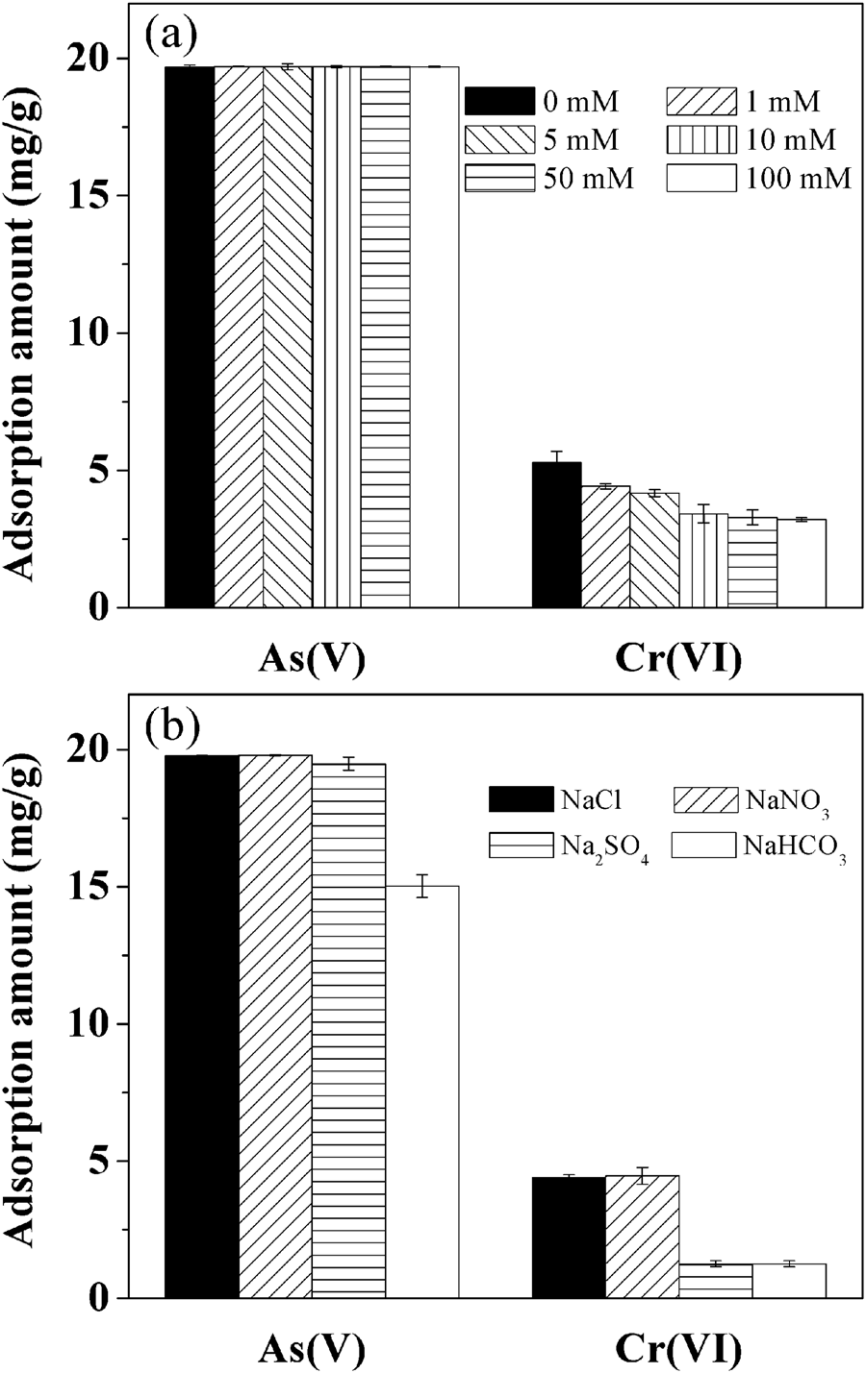
Effect of (**a**) ionic strength in NaCl background solution and (**b**) coexisting anions (1 mM) on As(V) and Cr(VI) adsorption on La-modified ceramic granules. Solution pH 6.8, As(V) concentration and adsorbent dosage were 20 mg/L and 1.0 g/L, and Cr(VI) concentration and adsorbent dosage were 3 mg/L and 0.5 g/L. The contact time was 24 h. Error bars represent standard deviations from triplicate experiments.

The influence of ionic strength on As(V) and Cr(VI) adsorption was investigated in NaCl solutions and corresponding results were shown in Fig. 9a. Nearly complete removal of As(V) (>98%) was observed in NaCl solutions with ionic strength ranging from 0 to 100 mM, indicating that ionic strength had little influence on As(V) adsorption. In contrast, Cr(VI) adsorption was more sensitive to ionic strength than As(V) that increasing the ionic strength gradually decreased the adsorption of Cr(VI). The observation for above As(V) and Cr(VI) adsorption trends suggested that their types of bonding interaction with adsorbents might be different and the outer-sphere complexation was likely to involve in Cr(VI) adsorption^45^.

The effects of a suite of coexisting anions that are commonly present in natural water matrices (Cl^−^, ${{\rm{NO}}}_{3}^{-}$, ${{\rm{SO}}}_{4}^{2-}$ and ${{\rm{HCO}}}_{3}^{-}$) on the As(V) and Cr(VI) adsorption were investigated under the same ion concentration (1 mM). As showed in Fig. 9b, the presence of Cl^−^, ${{\rm{NO}}}_{3}^{-}$ and ${{\rm{SO}}}_{4}^{2-}$ had negligible effects on the removal of As(V), while the adsorption amount of As(V) decreased by ~25% in the presence of ${{\rm{HCO}}}_{3}^{-}$, suggesting a mild inhibitory effect of ${{\rm{HCO}}}_{3}^{-}$ on As(V) adsorption. For Cr(VI) adsorption, Cl^−^ and ${{\rm{NO}}}_{3}^{-}$ also posed minimal effects on Cr(VI) removal, but strong inhibition was observed in the presence of ${{\rm{SO}}}_{4}^{2-}$ and ${{\rm{HCO}}}_{3}^{-}$. Similar observation for the effects of these four coexisting anions has been reported in previous studies examining the adsorption of As(V) and Cr(VI) by (hydrous) metal oxides^46,47^. ${{\rm{SO}}}_{4}^{2-}$ was reported to be adsorbed predominantly via outer-sphere complexation at pH higher than 6^48^, and thus the decrease of Cr(VI) adsorption with ${{\rm{SO}}}_{4}^{2-}$ indicated that ${{\rm{SO}}}_{4}^{2-}$ might compete with Cr(VI) for outer-sphere complexation with the surface sites. It is consistent with the decreasing trend of Cr(VI) adsorption with increasing ionic strengths that suggested the formation of outer-sphere complexes. At the same time, inhibitory effects of ${{\rm{HCO}}}_{3}^{-}$ on As(V) and Cr(VI) were probably due to the formation of inner-sphere complex or precipitate between ${{\rm{HCO}}}_{3}^{-}/{{\rm{CO}}}_{3}^{2-}$ and La compounds on the adsorbent surface, both of which may alter the sorbent surface properties^49^.

Based on the observation from batch adsorption experiments, combined with the solid characterization results, the adsorption of As(V) and Cr(VI) by the La-modified ceramic materials may be attributed to both electrostatic interaction and the formation of surface complexes between La surface functional groups and the anions. Different adsorption behaviors were found between Cr(VI) and As(V) on the La-modified ceramic material, and the material displayed higher sorption affinity for As(V) than Cr(VI). The observed difference in their adsorption behavior was likely caused by the distinct bonding types between anions and adsorbents, and more detailed mechanisms that govern the adsorption of As(V) and Cr(VI) worth further investigation.

## Conclusion

In this study, a cost-effective and environmentally friendly granular adsorbent was prepared by porous ceramic material and modified by lanthanum for removal of two challenging anionic waterborne pollutants As(V) and Cr(VI). The granular ceramic materials were synthesized using naturally abundant clay and recycled paper fiber. La modification was achieved through coating the ceramic surface with La(NO_3_)_3_ by thermal treatment. Coating temperature was critical for La modification and the subsequent As(V) and Cr(VI) adsorption. At the thermal temperature of 385 °C, the modified ceramic materials exhibited maximum adsorption capacities for both As(V) and Cr(VI) under environmentally relevant conditions. Furthermore, both As(V) and Cr(VI) adsorption kinetics followed pseudo-second order kinetic model, and Langmuir model can be used to describe their adsorption isotherms. The materials exhibited high removal efficiency of both As(V) and Cr(VI) under circumneutral conditions, and the performance decreased at basic conditions (pH > 9). Ionic strength and common coexisting anions including Cl^−^, ${{\rm{NO}}}_{3}^{-}$, ${{\rm{SO}}}_{4}^{2-}$ had little effects on As(V) adsorption, while the presence of ${{\rm{HCO}}}_{3}^{-}$ slightly inhibited As(V) adsorption. In contrast, the adsorption of Cr(VI) decreased with increasing ionic strength, and the presence of ${{\rm{HCO}}}_{3}^{-}$ and ${{\rm{SO}}}_{4}^{2-}$ showed inhibitory effect on Cr(V) adsorption. Furthermore, SEM, surface area measurement, TGA, zeta potential determination, and FTIR analysis were used to extensively characterized La-modified ceramic materials, and results suggested that both electrostatic interaction and complex formation with surface functional groups were involved in As(V) and Cr(VI) adsorption. LaONO_3_ and its associated ligand exchange products (e.g., LaOOH) have been identified as the primary La compounds formed at 385 °C that contributed to the adsorption of As(V) and Cr(VI). La-modified ceramic material exhibits as an effective, convenient, low-cost and sustainable adsorbent for anionic contaminant removal from drinking water sources, and it shows great promise for POU water purification system application, especially in developing countries. Future work will be dedicated to (1) investigating novel substrate material with high surface area for La modification and (2) elucidating the mechanisms that govern the removal of As(V) and Cr(VI).

## Methods

### Materials

Red art clay used in this study was obtained from Resco products Inc (USA). Its main chemical composition includes 64.2% SiO_2_, 16.4% Al_2_O_3_, 7% Fe_2_O_3_, 4.1% K_2_O, 1.6% and MgO (wt%), as provided by the manufacturer. Recycled paper fiber, used as pore maker in ceramic material fabrication, was obtained from local market. Clay and recycled paper fiber were both used as received. All chemicals, including Na_2_HAsO_4_·7H_2_O, Na_2_CrO_4_·4H_2_O, La(NO_3_)_3_·6H_2_O, HCl, NaOH, NaCl, NaNO_3_, Na_2_SO_4_ and NaHCO_3_ (Fisher Scientific, USA), are analytical grade and were used without further purification. Stock solutions (1000 mg/L) of As(V) and Cr(VI) were prepared by dissolving Na_2_HAsO_4_·7H_2_O and Na_2_CrO_4_·4H_2_O in water, respectively. As(V)/Cr(VI) working solutions were freshly prepared by diluting As(V)/Cr(VI) stock solutions. Ultrapure water (resistivity >18.0 MΩ) was used for all experiments.

### Preparation of unmodified ceramic granules

The ceramic materials used in this research were made of red art clay and recycled paper fiber. The red art clay was mixed with recycled paper fiber and water in the ratio of 10:1:5 (by weight). The homogenized mixture paste was molded into small cylindrical pieces using plastic pipes. The clay cylinders were air-dried at room temperature for 2 days and then fired in an electronic kiln (Olympic Kilns, USA). The temperature configuration for the kiln firing was: 1) increase at a rate of 60 °C/h from room temperature to 80 °C, holding for 3 h; and 2) increase at a rate of 150 °C/h to 900 °C, holding for 1 h. After being taken out of the kiln, the ceramic cylinders were broken into smaller blocks and sieved for the fraction of 18 to 45 mesh sizes. The sieved ceramic granules were cleaned repeatedly through ultrapure water rinsing, dried at 105 °C, and stored in plastic containers for characterization and further modification.

### Modification of granular ceramic material by lanthanum nitrate

La(NO_3_)_3_·6H_2_O was used as the precursor for the surface modification of ceramic granules. It is well established that, in the presence of ambient air, the thermal decomposition of La(NO_3_)_3_·6H_2_O proceeds through a series of steps that include dehydration, decomposition to LaONO_3_, La intermediate compounds and La_2_O_3_^18–20^. The thermal treatment of La(NO_3_)_3_·6H_2_O-amended ceramic materials under different temperatures thus allowed for the systematic investigation on the effects of different types of La(III) modification on the removal of As(V) and Cr(VI).

Granular ceramic material prepared above were firstly immersed in a saturated La(NO_3_)_3_ solution. The mixtures were then heated for 3 h in a furnace (Thermo Scientific, USA) at 300 °C, 385 °C, 500 °C and 800 °C, respectively. The treated ceramic granules were then cooled at room temperature and repeatedly rinsed with ultrapure water to remove any unstable or loosely attached La components. The modified ceramic granules were then dried in oven at 105 °C and stored in polypropylene bags before further use.

### Batch adsorption experiments

To fully investigate the Cr(VI) and As(V) adsorption behavior by La-modified ceramic material, batch experiments were conducted on a rotator (Techne TSB3, USA) at room temperature (22 ± 2 °C) with an initial pH of 6.8 to determine the adsorption kinetics and isotherms of As(V) and Cr(VI) by selected La-modified ceramic granules without pH adjustment. For adsorption kinetics, ceramic granules were mixed with As(V) (20 mg/L) or Cr(VI) (3 mg/L) solutions in centrifuge tubes. The ceramic granule loadings were 1.0 g/L and 0.5 g/L for As(V) and Cr(VI), respectively. At preselected times after the initiation of the adsorption experiments (e.g., 1 min, 5 min up to 48 hours), samples were collected, filtered immediately through a 0.22μm cellulose acetate filter and preserved for analysis. Adsorption of As(V) and Cr(VI) to the filters was confirmed negligible under experimental conditions. As(V) and Cr(VI) adsorption isotherms were obtained by varying the initial As(V) and Cr(VI) concentrations from 1.5 mg/L to 75 mg/L. A series of experiments were then performed to investigate the influence of ionic strength (0–100 mM, provided by NaCl) and coexisting anions that include chloride (Cl^−^), nitrate (${{\rm{NO}}}_{3}^{-}$), sulfate (${{\rm{SO}}}_{4}^{2-}$) and bicarbonate (${{\rm{HCO}}}_{3}^{-}$). Additionally, the effect of solution pH was tested by varying the initial solution pH from 4 to 11, with 0.1 mol/L HCl and 0.1 mol/L NaOH adjustment.

As(V) concentrations in the filtrates were measured by inductively coupled plasma atomic emission spectroscopy (ICP-AES, Perkin Elemer Optima 2100 DV, USA) after acidified by 2% HNO_3_ and Cr(VI) concentrations were determined by the 1,5-diphenylcarbazide method^50^. The quantify of adsorbed As(V) or Cr(VI), *q*_*e*_ (mg/g), was calculated by the following mass balance equation:

5 $${{\rm{q}}}_{{\rm{e}}}=\frac{({{\rm{C}}}_{{\rm{i}}}-{{\rm{C}}}_{{\rm{e}}}){\rm{V}}}{{\rm{W}}}$$

where *C*_*i*_ and *C*_*e*_ (mg/L) are the initial and final As(V) or Cr(VI) concentration in solution, respectively. *V* (L) is the volume of As(V) or Cr(VI) solution, and *W* (g) is the mass of ceramic materials. Results from the adsorption kinetics experiments indicated that adsorption equilibrium was generally reached within 24 h.

### Material characterization

The unmodified and La-modified ceramic materials were characterized to determine their morphological and physicochemical properties. SEM (Hitachi S-4800 FE-SEM, Japan) imaging was carried out to characterize the morphology of ceramic granules before and after La-modification. The La content in both the unmodified and La-modified ceramic granules was quantified by extracting the materials by acid digestion (2% HNO_3_), filtering the extraction liquid using 0.22-μm cellulose acetate filters, and measuring La concentration in the filtrates by ICP-AES. The specific surface area was measured via nitrogen adsorption through the BET method using a Micromeritics ASAP 2000 surface area analyzer (Micromeritics Co., USA). Attenuated Total Reflectance (ATR)-FTIR analysis was performed using a Bruker Vector 22 spectrometer (Bruker, Germany). The vibrations corresponding to the wavenumbers ranging from 650 to 4000 cm^−1^ were collected with the resolution of 4 cm^−1^. Zeta potential of adsorbents was measured by a Zetasizer Nano ZS90 (Malvern Instruments, UK). TGA was performed on a TA SDTQ650 (TA instruments, US) in the temperature range of 50 to 850 °C with an air flow rate of 100 mL/min and a heating rate of 10 °C/min.

## Supplementary information

Supplementary Information
