# Supplementary material for: Removal of Arsenate and Chromate by Lanthanum-modified Granular Ceramic Material: The Critical Role of Coating Temperature
Source: Sci Rep. 2019 May 22;9:7690. doi: 10.1038/s41598-019-44165-8 (PMC6531467; doi:10.1038/s41598-019-44165-8)
Supplement: Supplementary file 1 — Supplementary Information [file 41598_2019_44165_MOESM1_ESM.pdf]

Supplementary Information

Removal of Arsenate and Chromate by Lanthanum-modified Granular Ceramic Material: The Critical Role of Coating Temperature

Haiyan Yang[1, 2], Yin Wang[2*], John Bender[3], and Shangping Xu[1*]

[1]Department of Geosciences, University of Wisconsin—Milwaukee, Milwaukee, WI 53201, USA

[2]Department of Civil and Environmental Engineering, University of Wisconsin—Milwaukee, Milwaukee, WI 53201, USA

[3]Peck School of the Arts, University of Wisconsin—Milwaukee, Milwaukee, WI 53201, USA

*Corresponding author

Shangping Xu, 3209 N. Maryland Ave., Milwaukee, WI 53211. xus@uwm.edu, 414-229-6148

Yin Wang, 3200 N. Cramer St., Milwaukee, WI 53211. wang292@uwm.edu, 414-229-3137

NUMBER OF PAGES: 5

NUMBER OF TABLES: 3

NUMBER OF FIGURES: 1

Contents

Table S1 BET surface area and La content percentages of La-modified granular ceramic materials treated at different temperatures.

Table S2. Kinetics curve fitting parameters for adsorption of As(V) and Cr(VI) on La-modified granular ceramic adsorbents treated at 385 ºC.

Table S3. Comparison of adsorption of As(V) and Cr(VI) on various adsorbents.

Figure S1. As(V) and Cr(VI) adsorption amounts on ceramic granules modified with $La(NO_3)_3$ at different temperatures normalized by surface area of adsorbent.

Table S1 BET surface area and La content percentages of La-modified granular ceramic materials treated at different temperatures.

Sample	BET surface area ($m^2·g^{-1}$)	La content percentage (wt%)
w/o modification	2.79	ND[a]
300 °C	2.65	0.65±0.01
385 °C	5.24	20.4±0.6
500 °C	4.47	24.8±0.4
800 °C	6.44	25.8±0.5

[a] ND: not detected

Table S2 Kinetics curve fitting parameters for adsorption of As(V) and Cr(VI) on La-modified granular ceramic adsorbents treated at 385 ºC.

anion species	pseudo-first order kinetic model			pseudo-second order kinetic model		
	k_1 (h^{-1})	q_e ($mg·g^{-1}$)	r^2	k_2 ($g·mg^{-1}·h^{-1}$)	q_e ($mg·g^{-1}$)	r^2
As(V)	3.18±0.50	18.7±0.8	0.902	0.233±0.035	19.8±0.63	0.963
Cr(VI)	0.182±0.016	5.74±0.15	0.981	0.0328±0.003	6.68±0.12	0.995

Table S3 Comparison of adsorption of As(V) and Cr(VI) on various adsorbents.

Sorbates	Adsorbents	solution pH	adsorption capacity ($mg \cdot g^{-1}$)	reference
As(V)	Fe-impregnated ceramic	6.9	8.49	1
	chitosan/clay/magnetite	5.0	5.9	2
	La-impregnated silica gel	7.0	3.75	3
	magnetite-maghemite nanoparticles	5	10.6	4
	La-modified ceramic	6.8	22.9	this study
Cr(VI)	Bituminous coal	5.0-8.0	7.0	5
	CTAB modified silica gelatin	5.8	5.8	6
	Alkyl ammonium surfactant bentonite	5.0	8.36	7
	red clay modified by HDTMA	5.5	4.47	8
	La-modified ceramic	6.8	13.0	this study

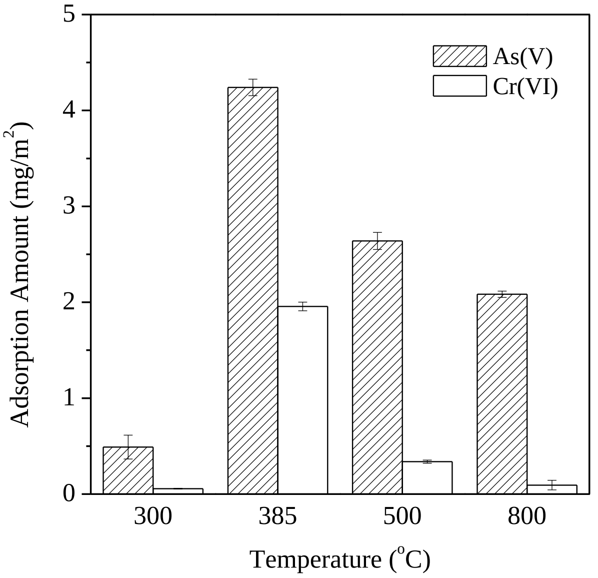

Figure S1 As(V) and Cr(VI) adsorption amounts on ceramic granules modified with $La(NO_3)_3$ at different temperatures normalized by surface area of adsorbent.

Reference

1 Chen, R., Zhang, Z., Lei, Z. & Sugiura, N. Preparation of iron-impregnated tablet ceramic adsorbent for arsenate removal from aqueous solutions. *Desalination* **286**, 56-62 (2012).

2 Cho, D.-W. *et al.* A novel chitosan/clay/magnetite composite for adsorption of Cu (II) and As (V). *Chem. Eng. J.* **200**, 654-662 (2012).

3 Wasay, S. A., Haron, J. & Tokunaga, S. Adsorption of fluoride, phosphate, and arsenate ions on lanthanum impregnated silica gel. *Water Environ. Res.* **68**, 295-300, (1996).

4 Chowdhury, S. R. & Yanful, E. K. Arsenic and chromium removal by mixed magnetite-maghemite nanoparticles and the effect of phosphate on removal. *J Environ Manage* **91**, 2238-2247, doi:10.1016/j.jenvman.2010.06.003 (2010).

5 Di Natale, F., Lancia, A., Molino, A. & Musmarra, D. Removal of chromium ions form aqueous solutions by adsorption on activated carbon and char. *J. Hazard. Mater.* **145**, 381-390 (2007).

6 Showkat, A. M. *et al.* Analysis of heavy metal toxic ions by adsorption onto amino-functionalized ordered mesoporous silica. *Bull. Korean Chem. Soc.* **28**, 1985 (2007).

7 Sarkar, B. *et al.* Remediation of hexavalent chromium through adsorption by bentonite based Arquad® 2HT-75 organoclays. *J. Hazard. Mater.* **183**, 87-97 (2010).

8 Gładysz-Płaska, A., Majdan, M., Pikus, S. & Sternik, D. Simultaneous adsorption of chromium (VI) and phenol on natural red clay modified by HDTMA. *Chem. Eng. J.* **179**, 140-150 (2012).
